# Comparative Analysis of Coatings Applied for Anti-Corrosion Protection of Public Transport Vehicles’ Structural Parts

**DOI:** 10.3390/ma17153763

**Published:** 2024-07-30

**Authors:** Wojciech Skotnicki, Dariusz Jędrzejczyk

**Affiliations:** Faculty of Mechanical Engineering and Computer Science, University of Bielsko-Biala, Willowa 2, 43-309 Bielsko-Biala, Poland; djedrzejczyk@ubb.edu.pl

**Keywords:** corrosion resistance, anti-corrosion coatings, zinc galvanization, public transport vehicles

## Abstract

The conducted research focused on anti-corrosion systems applied for the protection of structural parts used in public transport vehicles. Detailed tests were carried out on samples taken from the brackets supporting the doors of a public transport bus. This work includes the results of the chemical analysis of the composition of snow–mud samples taken from the selected bus route and the results of laboratory tests performed on samples with various anti-corrosion coatings. Four types of samples made of S235JR steel with a zinc coating deposited by thermo-diffusion, electroplating, hot-dip zinc galvanization, and the cataphoresis method were tested. Both non-destructive tests—NDTs (the measurement of coating thickness and roughness, microscopic observations)—and destructive tests—DTs (scratch tests, salt chamber tests)—were performed. The conducted tests proved that the most effective method is the use of anti-corrosive hot-dip zinc coating.

## 1. Introduction

The cost of corrosion is a growing global problem—for example, in 2014, in China, its value was approximately CNY 2127.8 billion (USD ~310 billion), which constituted about 3.34% of the gross domestic product [1]. Corrosion affects many parts of cars, reducing their value and decreasing the strength of structures [2,3]. Because public transport vehicles move in different administrative (cities, villages, and industrial areas) and climatic (dry, temperate, and continental) zones, it is very difficult to choose a universal anti-corrosion protection system for them. For example, a bus traveling in coastal areas is exposed to aerosols containing chloride ions, which accelerate corrosion processes [4]. The main corrosion hazard for vehicles that regularly drive through cities located at the foots of mountains is mainly snow–mud with high road salt content. A temperate, warm, and transitional climate promotes the development of corrosion that affects the steel structures of many vehicles. Although one paper [4] states that the proper maintenance of a bus body ensures the long-term and reliable operation of the bus, other data confirm that, after 5–9 years of bus operation, structural corrosion makes it impossible to continue to operate it [5,6]. Some authors [7] even propose the use of stainless steel in bus structures, which could significantly improve durability. 

A similar corrosion problem applies to both public and private transport vehicles. In August 2016, Mazda confirmed that more than 190,000 CX-7 crossovers from the model years 2007–2012 had to be recalled due to the risk of the corrosion of front suspension ball joint fittings [8]. The problem was related to ball joint corrosion in snowy conditions and the possibility of losing steering control. A similar action was also announced by Honda: nearly 564,000 components of its older model—CR-V [9]—were recalled because of road salt corrosion to the frame, which meant there was a risk that parts of the rear suspension would fall off. 

The situation in Poland is specific—in most cases, public transport vehicles are not garaged (they are parked directly in an unroofed space—the depot). The protection of components against corrosion is a great challenge, especially if we take into account that mostly used/old buses are imported to Poland. According to the report in [10], it can be stated that, at the end of 2022, there were 66.5% (2298 units) more used buses than new ones. For example, in February 2023, 47% of the total number of imported public transport vehicles were manufactured between 2005 and 2009. Buses manufactured from 2010 to 2019 (42%) were in second place, and buses manufactured from 2000 to 2004 (8%) were in third place. 

The corrosion rate of structural elements is influenced by several factors, such as climate (humidity, temperature [11]), mechanical load, heat loads, and environment pollution (the chemical composition of atmospheric water [12,13] and other aspects). Aggressive environments areas are defined in the PN-EN ISO 12944-4:2018-2 [14] standard.

It was found in paper [15] that, although zinc and cataphoretic coatings are most commonly used for corrosion protection, existing technologies should be improved. The authors suggest the application of new polymeric materials and optimal treatment methods considering the following relation: rate of corrosion/bus lifetime. The Life Cycle Cost (LCC) analysis made in paper [16] confirmed the long-term financial benefits to the user of using corrosion protection only in the form of a zinc coating made by hot-dip galvanization technology. Because of its relatively low price [17], zinc is the most commonly used element in the production of anti-corrosion coatings.

Generally, zinc coatings applied to different structure elements can be produced by four methods: hot-dip galvanization, electro-galvanization, zinc lamella, and sherardization (thermal diffusion) [18,19,20].

The corrosion resistance of the coatings mentioned above varies in a wide range depending on the conditions of the experiment [21,22,23,24]. Opinions are very divided; some authors [21,22] confirmed the higher corrosion resistance of thermo-diffusion coatings in comparison to hot-dip ones. Next, in paper [23], a thermo-diffusion-protected steel sheet was less resistant to corrosion than hot-dip zinc-galvanized material. Under some conditions, Zn coatings (galvanic, hot-dip, and thermo-diffusion) showed similar corrosion resistance [24]. Zinc coatings very often compete with cataphoretic coatings, which are used frequently in the automotive industry and have a very wide range of corrosion resistance—up to 1000 h [25]. A big disadvantage of cataphoretic coatings is their low resistance to UV rays—<100 h [25].

In addition to the significant variation in corrosion resistance, the analyzed coatings still differ in terms of thickness and mechanical properties. The hardness measured on the surface of zinc treated with a typical hot-dip coating is close to the value of 50 HV [26,27,28]. The heat treatment of dip-coated zinc allows its hardness to increase to 200 HV [29,30].

**Figure 1:**
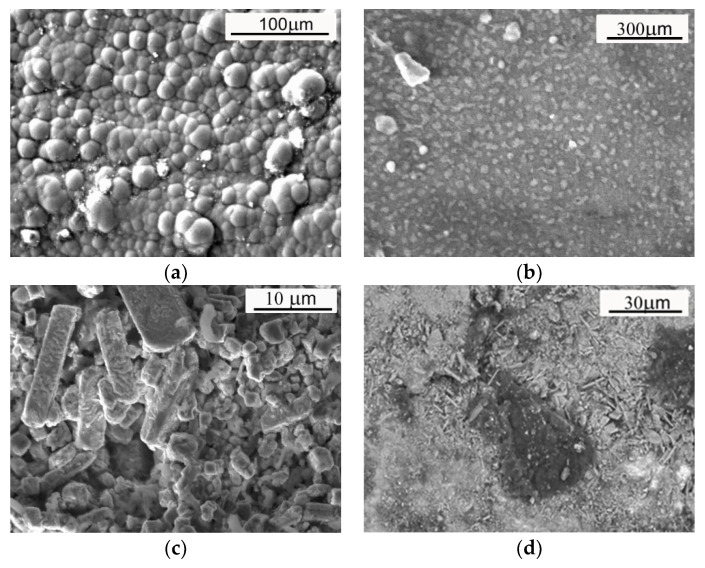
Outer surface appearance of the zinc coatings observed under the scanning microscope; (**a**) galvanic coating; (**b**) hot-dip coating; (**c**) heat-treated hot-dip coating; (**d**) sherardized coating (own investigation).

The main factors influencing the anti-corrosion properties of zinc coatings are their microstructure [31,32] and thickness [32,33,34,35]. The microstructures of hot-dip and thermo-diffusion coatings are composed of phases occurring in the Fe-Zn equilibrium system: η, ζ, δ, Г_1_, and Г [27,36,37]. Usually, the outer layer has a decisive influence on the anti-corrosion properties of a coating. The appearance of the outer surfaces of zinc coatings observed under a scanning microscope is presented in Figure 1. In the case of a hot-dip coating, the outer layer is an iron solid solution in zinc—η—which is formed during production on the outer surface as it is pulled out of the bath. The coating microstructure deposited after thermal diffusion is similar to that of the hot-dip zinc coating, but instead of an η phase in the outer layer, a mixture of ζ and zinc [21], a ζ-FeZn_13_ phase [22] or (Г + δ) phases exist [38]. The hardness of the tested coatings is a derivative of the properties of individual phases [20,21]. The hardness values measured by Pokorny [37] show that the δ phase is generally about 10% harder than the Г phase—the obtained hardness values of the δ phase were even in the range 330 to 460 HV. According to the data presented in [20,39], the δ phase in TD coating is about 15% harder than the Γ phase. The hardness of the hot-dip zinc coating after heat treatment reaches ab. 200 HV because a columnar phase ζ appears on the outer surface—Figure 1c. The same mechanism makes the outer layer of the thermo-diffusion coating much harder (ab. 300 HB) than that of a hot-dip coating. The hardness of galvanic zinc coating ranges from 40 to 60 HV [40]. The tribological properties are directly correlated with the hardness and microstructure of the applied coating.

Considering the diversity of anti-corrosion properties of the analyzed protective coatings, the aim of the presented research was to verify the most commonly used systems in terms of protecting the structural parts of buses used in strictly defined and particularly difficult conditions (such as during the winter season, with mud and snow). 

The most novel aspect of the presented article is corrosion tests conducted in conditions corresponding to real corrosive environment, using test samples cut from an actual structural element —the bracket supporting the bus doors. The mechanical resistance of the coatings was assessed for the first time using the Erichsen Scratch Hardness Tester 413, produced by Erichsen, Hemer, Germany. Moreover, the kinetics of the corrosion processes, expressed as the percentage of surface covered by “red corrosion”, was measured very precisely using computer image analysis—ImageJ software version 1.54i.

## 2. Materials and Methods

**Figure 2:**
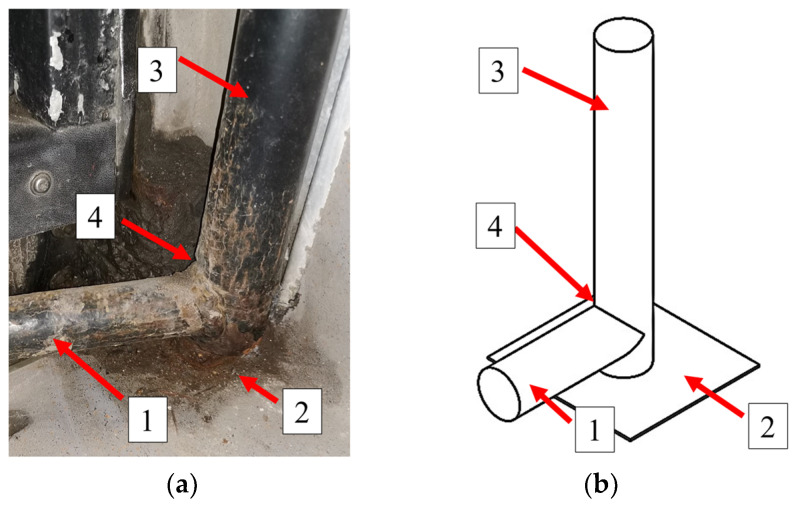
Appearance of the analyzed bracket during operation: Leader 9LE Autosan (**a**); bracket construction drawing (**b**); 1—bracket; 2—bus floor; 3—steel tube; 4—welded connection.

During the tests, four coatings were compared: zinc coatings created by thermo-diffusion, electroplating, hot-dip zinc galvanizing, and coating applied with the cataphoresis method. These coatings were deposited on the samples cut from the new (unused) bracket supporting the bus doors, made of S235JR steel (Figure 2). The bracket structure is composed of two pipes (1 and 3) connected to each other by fillet welds (4). 

The chemical composition (by weight percentage) of the structural steel S235JR used was as follows: 0.18% C, 1.45% Mn, 0.036% P, 0.037% S, 0.43% Cu. The balance was Fe. The properties of the coatings were tested on samples with dimensions of 50 mm × 50 mm × 2 mm. The surface of the samples was cleaned mechanically through abrasive blasting. The tested coatings were applied to the steel surface in industrial conditions in accordance with the methodology presented in Table 1.

The study includes the measurement of the following parameters:

- TDSs value (total dissolved solids) of snow–mud—an indicator determining the total content of all mobile charged ions in an aqueous solution (such as salts and minerals dissolved in water). This parameter was measured using a laboratory conductivity meter, inoLab Cond Level 2, produced by CAMLogic, Cavrriago, Italy.
- Snow–mud pH value using a microprocessor tester pH/mV/temp/RS232 produced by LPP Equipment, Swiecice, Poland.
- Surface roughness (the microscopic non-contact attachment—Phase View system produced by PhaseView, Verrieres Le Buisson, France with ZeeScan software version 2.4).
- Coating thickness (PosiTector 6000MP magnetic induction tester with 90° depth finder—DeFelsko New York, NY, USA).
- Coating corrosion resistance: Evaluated using an Ascott CC1000iP salt chamber produced by Ascott, Staffordshire, UK. The tests were conducted in accordance with the PN-EN ISO 9227:2017-06 standard [45], using a corrosive medium of NaCl at 50 ± 5 g/dm^3^, with a solution density of 1.035 g/cm^3^ and fall value of 1.033 g/cm^3^, pH 6.7, air pressure of 1 bar, and a chamber temperature of 35 °C. After testing, parts were cleaned in an aqueous solution of 12% HCL with the addition of a 0.1% corrosion inhibitor PICKLANE 50 produced by COVENTYA, Weiland, Germany.
- Scratch resistance: tested using the Erichsen Scratch Hardness Tester 413 produced by Erichsen, Hemer, Germany.

## 3. Results and Discussion

### 3.1. Measurement of TDS (Total Dissolved Solids) of Snow–Mud

**Figure 3:**
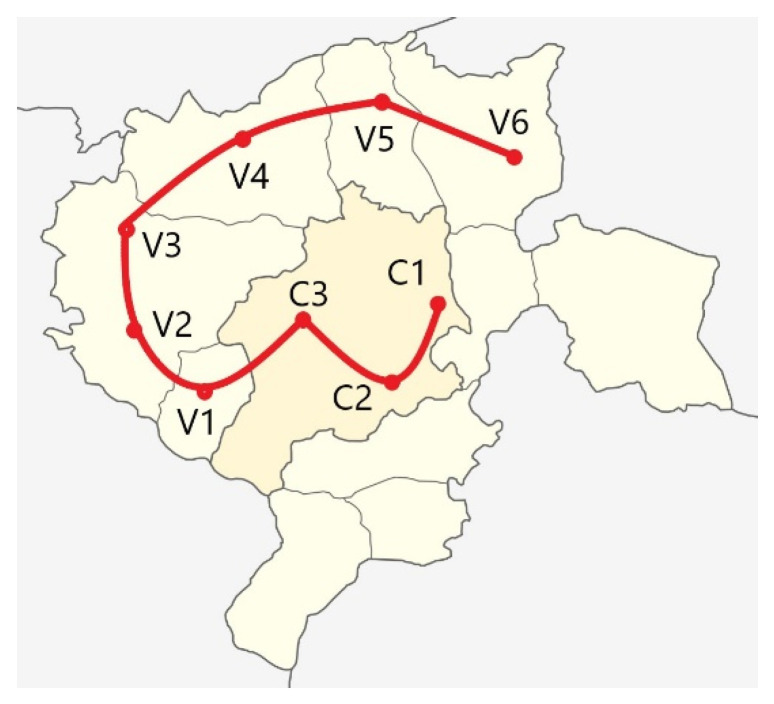
Bus route (red line) with marked places where samples were taken (red circle).

To determine the chemical composition of the corrosive environment for testing, slush samples were taken from the places situated on a chosen bus route. This route is considered one of the most representative for the studied region (southern part of Poland). The bus travels in several zones: urban, rural, and partly industrial. In addition, the bus stops are situated in diverse environments, i.e., some of them are located in places that are regularly cleared of snow, while others are covered with a mixture of snow, mud, salt, and dirt. Samples (three for each) were taken from nine locations, including three in the city and six in rural and industrial areas, as illustrated in Figure 3. 

**Figure 4:**
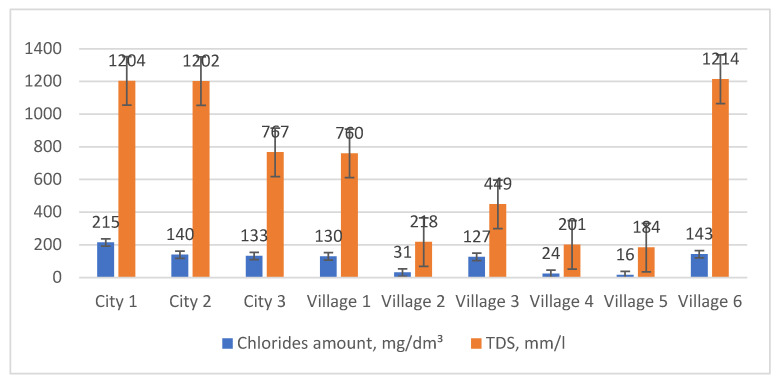
Amounts of dissolved solids in the tested samples.

The conducted measurements (TDS, chloride amounts, conductivity, and pH—Figure 4, Table 2) indicate that the highest chlorides content in slush (133–215 mg/dm^3^) occurs in urban areas. The percentage of chlorides in relation to the total amount of dissolved substances TDS ranges from 9 to 28.3%, depending on the sampling area, with an average of 15.5% for the entire route (the average for the entire route is 15.5%). The conductivity of individual samples ranges from 447 to 2710 μS/cm, with the highest values measured in the area of the city and central areas of smaller villages (1855 to 2710 μS/cm). An increase in the content of dissolved solids corresponds to an increase in the specific conductivity of the water. Considering the correlation between corrosion rate and the specific conductivity of water [46], it can be assumed that the city area and village centers V1, V3, and V6 are the regions where the corrosion rate is likely to be the highest, due to specific conductivity levels ranging from 1096 to 2710 μS/cm. Soft and acidic waters, which contain both dissolved oxygen and other substances, are characterized by high aggressiveness towards metals [46]. According to Table 2, that the pH of all nine samples is within the range of 6.23 to 7.04 pH. The pH values suggest that different types of corrosion can be expected in the investigated area, as a pH below 6.5 typically favors uniform corrosion, while a pH of 6.5–8.0 promotes pitting corrosion.

### 3.2. Measurement of Coatings’ Thickness and Roughness

Measurements of the thicknesses of the coatings deposited on tested samples with dimensions of 50 mm × 50 mm × 2 mm were made by the magnetic method, in accordance with PN-EN ISO 2178:2016-06 [47]. Ten measurements were taken on each sample, and the results were averaged. The measured average coating thickness (with standard deviation) for tested samples was as follows: 30.7 µm (s.d. 2.54 µm) for the thermo-diffusion zinc coating; 13.4 µm (s.d. 0.16 µm) for the galvanic zinc coating; 45 µm (s.d. 0.98 µm) for the cataphoretic coating; 76.6 µm (s.d. 4.5 µm) for the hot-dip zinc coating. The measured coating thicknesses are within the usual applied ranges [48,49]: sherardized (10–75 µm), galvanized (8–25 µm), cataphoretic (15–50 µm), and hot-dip zinc coating (50–200 µm). 

The conducted measurements show that the thickest and most heterogeneous coating, as indicated by the highest standard deviation, was the hot-dip zinc coating. The lowest coating thickness was observed in the electro-galvanized samples, measuring 13.4 μm. The large variation in the thickness of the hot-dip zinc layer is attributed to the challenges of applying the coating process under industrial conditions, particularly with relatively small samples. The thickness of the tested paint coatings is very similar to that of sherardized coatings.

The measurements of the roughness of samples with different anti-corrosion coatings were carried out using the PhaseView system and the Axiovert 100A optical microscope (ZEISS, Oberkochen, Germany). The images were analyzed with GetPhase Reader software version 2.4. For each coating, roughness profiles, 2D surface topography, and 3D topography with a groove worn in the scratch test were determined. Ten measurements were made for each coating. The following parameters were measured:

- Ra: the arithmetic mean of all absolute roughness profile deviations from the centerline within the measurement length.
- Rz: the average absolute peak-to-valley height over five sequential sampling lengths within the measurement length.
- Rt: the total profile height.
- Sa: the extension of Ra to a surface, representing the arithmetic mean height over a defined area.

**Figure 5:**
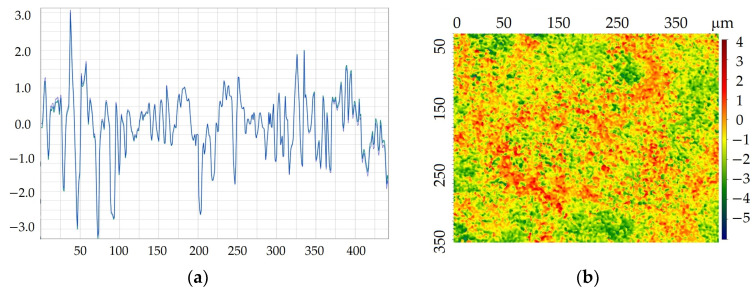
Example of a roughness profile (**a**) and surface topography for a cataphoretic coating (**b**).

Table 3 contains the specific measurements of these parameters. Figure 5 presents an example of a roughness profile and surface topography for a cataphoretic coating. 

The cataphoretic coating shows the lowest roughness (Ra = 0.64 µm). The electro-galvanized surface, with an Ra of 1.43 µm, is the most similar to a paint coating in terms of measured parameters. Among all the coatings tested, the hot-dip galvanized coating appears to be the worst (Ra = 2.14 µm). The visual aesthetics of the surface, quantified by the parameter Rz, was also the lowest for the hot-dip zinc coating. In contrast, the galvanic coating had the smoothest surface, as indicated by the smallest Rz value. These results are consistent with findings reported in other studies [49,50]. The tests were carried out in accordance with PN-EN ISO 21920 [51].

### 3.3. Results of Coatings’ Scratch Test

The coatings of bus brackets are particularly exposed to mechanical damage which can lead to fatigue cracking, erosion, hydro-erosion, and the abrasive wear of the protective layers [52,53]. In the present research, the resistance of coatings to mechanical damage was determined using Erichsen Scratch Hardness Tester 413 [54]. During the test, the specimen is clamped onto a turntable, while the test tool is fixed on a load arm. Both the arm length and the load can be adjusted to individual requirements with a load range of 0–10 N in 0.1 N steps and 0–1 N in 0.01 N steps. As the turntable rotates, the test tip presses against the coating surface, causing the abrasion of the outer layer. The following constant parameters were used: number of disc rotations—10; loading force—10 N; arm length—100 mm. The procedure involved several stages: measurement of the sample mass on an electronic scale before the test (with an accuracy of 0.001 g), performing a scratch test, measurement of the sample weight after the test, and conducting microscopic observations.

**Figure 6:**
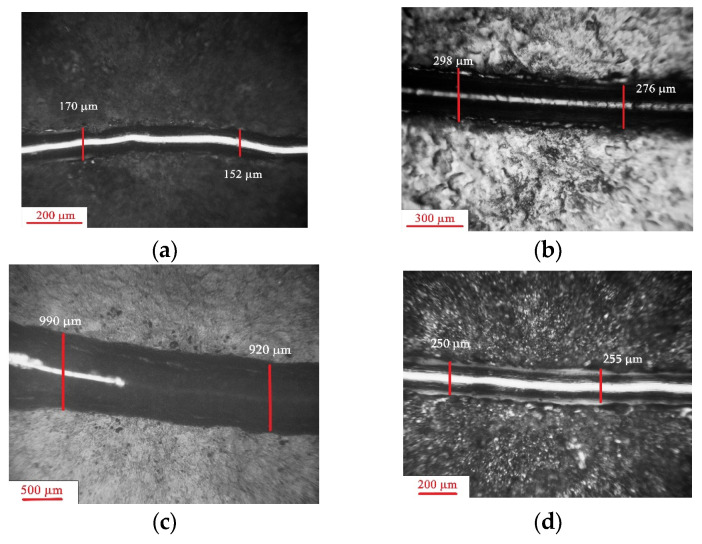
View of the groove worn during the scratch test—optical microscope: (**a**) thermo-diffusion coating; (**b**) galvanic coating; (**c**) cataphoretic coating; (**d**) hot-dip zinc coating.

**Figure 7:**
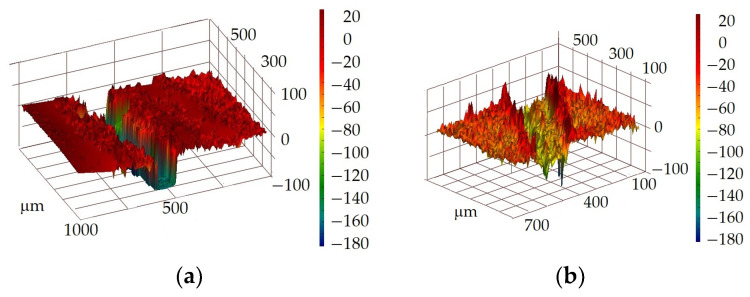
Example of surface topography observed near a worn groove—phase view attachment: (**a**) cataphoretic coating; (**b**) hot-dip zinc coating.

The appearance of the worn grooves is shown in Figure 6 and Figure 7. The width of the worn groove was as follows: 160.75 μm for the thermo-diffusion coating, 287.75 μm for the galvanic coating, 953.3 μm for the cataphoretic coating, and 250.5 μm for the hot-dip zinc coating. The tribological properties of the analysed coatings are correlated with its micro-structure and mechanical properties (hardness) of individual phases existing in the coating cross-section. According to the data in the literature, the highest hardness (ab. 300 HB [39]) is measured in the outer layer of thermo-diffusion coating. Phase η, observed in hot-dip zinc coating, shows hardness of about 50 HV [26,27,28], while the hardness of galvanic coating does not exceed 60 HV [40]. The hardness of the cataphoretic coating, as measured in [48], corresponds to a Vickers hardness value of 30 HV. The shape of the groove worn in the paint coating differs from that of the zinc coatings, being more regular with relatively smooth walls (Figure 7a). In contrast, the grooves in the thermo-diffusion and hot-dip coatings are irregular (Figure 7b), with outlines that significantly deviate from a regular curvature, and their walls are rougher. It was observed during the research that, in these cases, the indenter moved with much more resistance.

### 3.4. Corrosion Resistance Tests

**Figure 8:**
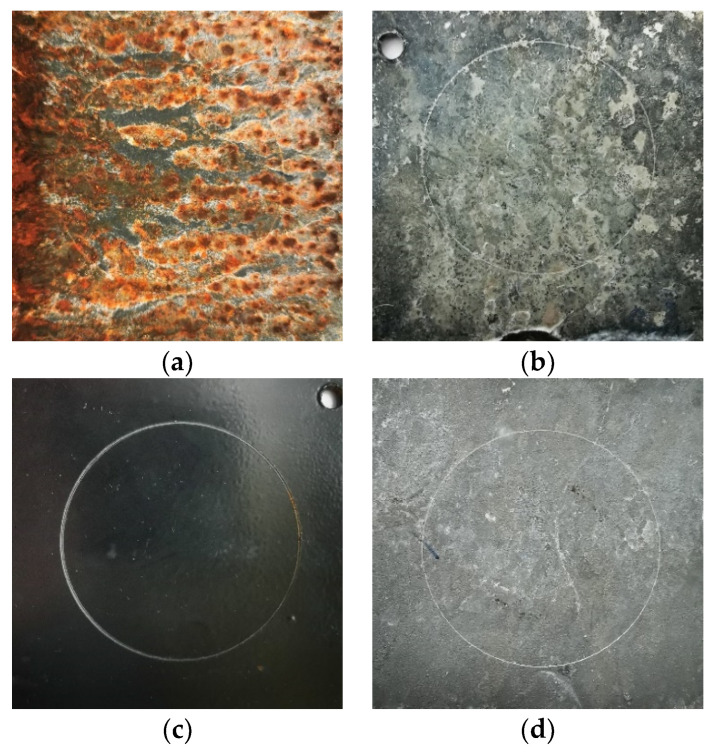
Surface appearance of the tested samples after the salt spray test (148 h): (**a**) thermo-diffusion coating; (**b**) galvanic coating; (**c**) cataphoretic coating; (**d**) hot-dip zinc coating.

The test lasted 1000 h, with the surface of the samples assessed at regular intervals of 24 h, in accordance with the standard. The appearance of the coatings surfaces after 148 h of corrosion tests is shown in Figure 8.

**Figure 9:**
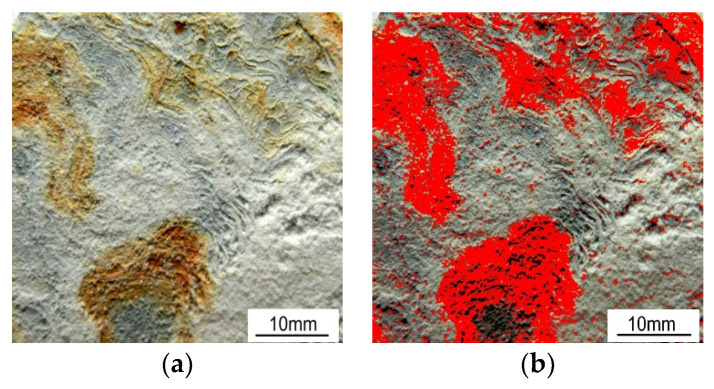
Example of the measurement of the red corrosion covered area using ImageJ software: (**a**) hot-dip zinc coating after 1000 h of NSS test—original image; (**b**) the same image, with red used to indicate the corroded/measured area—24%.

**Figure 10:**
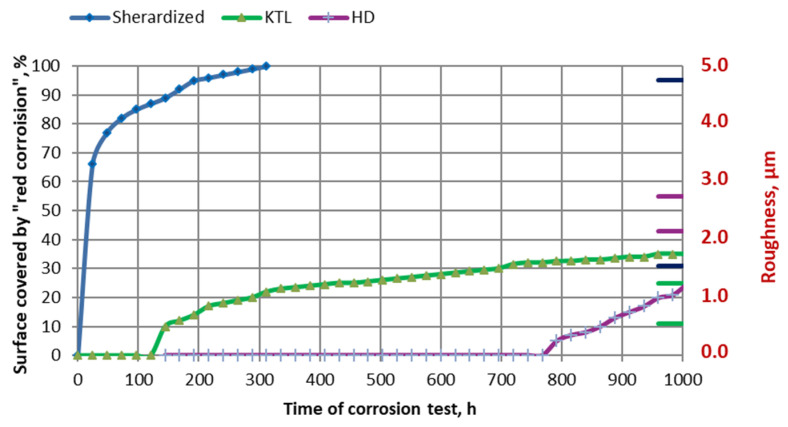
The kinetics of corrosion processes, expressed as the percentage of surface covered by “red corrosion” were measured using ImageJ software. Horizontal markers placed on the right side indicate the range of roughness changes observed during the test.

The extent of the area covered by red corrosion was determined using computer image analysis with ImageJ software. An example of such an analysis, specifically for the hot-dip zinc coating, is shown in Figure 9. A summary of the kinetics of the corrosion process observed on the surface of the samples together with the markers of the surface, along with markers indicating surface roughness (upper and lower values), is presented in Figure 10. The first signs of red corrosion appeared after 24 h on a sample covered with a thermos-diffusion coating. This result is entirely consistent with the research presented in [49,50]. As the test continued, both the area covered by red corrosion and the surface roughness increased. Red corrosion traces appeared on the cataphoretic coating (KTL) after 144 h, while such corrosion was noted on the hot-dip zinc coating after only 792 hours; red corrosion was observed on the galvanic coating during the test; hence, the kinetics of the galvanic coating process are not included in Figure 10. 

The trend toward increasing roughness as the NSS test progressed was the case for all the coatings. The final roughness values after the test were as follows:

- Cataphoretic coating—1.30 µm (an increase of approximately 0.66 µm);
- Hot-dip zinc coating—2.75 µm (an increase of approximately 0.61 µm);
- Thermo-diffusion coating—4.72 µm (an increase of approximately 3.01 µm);
- Galvanic coating—1.62 µm (an increase of approximately 0.19 µm).

## 4. Conclusions

1. The corrosive environment in which public transport operates during the winter season is characterized by a pH of 6.23–7.04 and chloride content of 133–215 mg/dm^3^; this places significant demands on the need for effective anti-corrosion systems. A simple cataphoretic coating alone is insufficient in this context.
2. Based on the results of the conducted experiment, it can be concluded that hot-dip zinc coating provides a much better solution for the anti-corrosion protection of bus door brackets compared to the cataphoretic coating previously used.
3. Although the corrosion resistance of the galvanic coating is the highest, with more than 1000 h until the appearance of red corrosion in the NSS test, its lower resistance to mechanical damage, combined with the relatively small thickness of the coating, may reduce its overall durability.
4. Very low corrosion resistance of the thermo-diffusion coating, which exhibited red corrosion after just 24 h in the NSS test, decisively eliminates it as anti-corrosion protection of the responsible structural parts.
5. Considering aesthetic aspects and the corrosive environment tested, the best solution for protecting visible structural elements would be a duplex coating, combining a hot-dip zinc coating with an outer cataphoretic layer.
6. Measurements of the roughness of corrosion-resistant coatings indicate that this parameter accurately describes the kinetics of the corrosion processes.

## Figures and Tables

**Table 1:** Methodology of deposition of tested coatings.

Sample No.	Kind of Coating	Sample Preparation Methodology
1	Thermo-diffusion according to PN-EN ISO 17668 [41]	Etching of the sample in 12% HCl solution; galvanizing in the powder with filler and activator (rotary chamber: 5–10 rpm, temperature: 400 °C, time: 4 h); cooling with air up to 25 °C.
2	Galvanized according to PN-EN ISO 2081 [42]	Chemical degreasing at 60 °C; etching in solutions: 18% HCl and 10% H_2_SO_4_ with inhibitors ACTANE 4200 produced by MacDermid Enthone, US; degreasing and electro-polishing at 60 °C and current 1000 A; electrolytic galvanizing in a Zn bath at a temperature of 35 °C; passivation with Cr^3+^, Co^2+^, NO_3_ ions at 45 °C (solution with a 1.9 pH).
3	Cataphoretic painting according to PN-EN ISO 12944-2:2018-02 [43]	Sample etching in 12% HCl solution, dipping in paint Cathoprime QT82-9436—water-soluble paint produced by BASF Coating AG, Münster, Germany (5.8–6.5 pH, voltage 230–270 V, time: 180 s), drying the coating for 4 h at 180 °C.
4	Hot-dip galvanized according to PN-EN ISO 10684:2006 [44]	Sample etching in 12% HCl solution; fluxing; hot-dip galvanizing at 460 °C in Zn bath with Al, Bi, Ni additives; cooling in water.

**Table 2:** TDS and pH of snow–mud measurement results.

Place of Sample Collection	TDS, mg/L	Chlorides Amount, mg/dm^3^	Percentage of Chlorides, %	Conductivity, μS/cm	pH
City—1	1204	215	17.9	2200	6.44
City—2	1202	140	11.7	2700	6.23
City—3	767	133	17.3	1874	6.69
Village 1	760	130	17.1	1855	6.85
Village 2	218	31	14.2	531	6.96
Village 3	449	127	28.3	1096	7.04
Village 4	201	24	12.1	488	7.01
Village 5	184	16	9.0	447	6.98
Village 6	1214	143	11.8	2710	7.00

**Table 3:** Selected parameters characterizing the surface condition of the tested coatings (s.d.—standard deviation).

Kind of Coating	Ra, μm (s.d.)	Rz, μm (s.d.)	Rt, μm (s.d.)	Sa, μm (s.d.)
Thermo-diffusion	1.71 (0.160)	6.05 (0.050)	10.24 (0.112)	2.49 (0.140)
Galvanic	1.43 (0.008)	3.86 (0.024)	7.54 (0.120)	1.40 (0.009)
Cataphoretic	0.64 (0.005)	4.01 (0.030)	6.07 (0.105)	0.64 (0.003)
Hot-dip	2.14 (0.022)	8.71 (0.091)	10.76 (0.155)	3.19 (0.055)

## Data Availability

The raw data supporting the conclusions of this article will be made available by the authors on request.
